# Converting Ni-loaded biochars into supercapacitors: Implication on the reuse of exhausted carbonaceous sorbents

**DOI:** 10.1038/srep41523

**Published:** 2017-01-27

**Authors:** Yifan Wang, Yue Zhang, Lei Pei, Diwen Ying, Xiaoyun Xu, Ling Zhao, Jinping Jia, Xinde Cao

**Affiliations:** 1 School of Environmental Science and Engineering, Shanghai Jiao Tong University, Shanghai 200240, China

## Abstract

Biochar derived from waste biomass has proven as a promising sorbent for removal of heavy metals from wastewater. However, proper disposal of such a heavy metal-containing biochar is challengeable. The major objective of this study is to create a reuse way by converting the heavy metal-loaded biochar into supercapacitor. Two biochars were produced from dairy manure and sewage sludge, respectively, and subjected to sorption of Ni from solution, and then the Ni-loaded biochar underwent microwave treatments for fabrication of supercapacitor. The specific capacitance of biochar supercapacitor increased with Ni loading, especially the Ni-loaded biochar further treated with microwave in which the capacitance increased by over 2 times, compared to the original biochar supercapacitors. The increase of capacitance in the Ni-loaded biochar supercapacitor following microwave treatment was mainly attributed to the conversion of Ni into NiO and NiOOH, which was evidenced by X-ray diffraction and X-ray photoelectron spectroscopy. The biochar supercapacitors, especially microwave-treated Ni-loaded biochar supercapacitors exhibited the high stability of specific capacitance, with less than 2% loss after 1000 charge-discharge cycles. This study demonstrated that Ni-loaded biochar can be further utilized for generation of supercapacitor, providing a potential way for the reuse of exhausted carbonaceous sorbents.

Biochar is a carbonaceous solid material produced through pyrolysis of the waste biomass such as manure, wood chips, and sewage sludge^1^,^2^,^3^. Therefore, biochar is rich in oxygenated groups, such as carboxyl, hydroxyl, and phenolic surface function groups and has meaningful properties such as stability, low cost, porosity, nontoxicity, and sustainability^4^. These specific structure and properties contribute to the wide application of biochar. For example, biochar shows a great capacity for sorbing heavy metals such as Ni^2+^, Cd^2+^, Co^2+^, and Pb^2+^ in the aqueous solution. Liu *et al*. used *Phragmites australis*-derived biochar as the sorbent for sorption of Ni(II) from the aqueous solution, obtaining a sorption capacity of 19.2 mg g^−1^ 5. The biochar produced from broiler litter manure could sorb 59.7 mg g^−1^ Pb^2+^, 2.02 mg g^−1^ Cd^2+^, 16.5 mg g^−1^ Cu^2+^, and 1.76 mg g^−1^ Ni^2+^ 6. The heavy metals retained in the biochar are difficult to be desorbed, which makes the sorbent become hazardous waste if they are not well disposed^7^. Therefore, how to properly deal with the metal-loaded biochar may become a challenge.

Many studies have been done by using carbonaceous materials such as activated carbon and carbon nanotube as electrodes for microbial fuel cell and supercapacitors^8^,^9^,^10^. Supercapacitor, an essential electric device for energy storage, is undergoing rapid development with its potentially broad applications in automobiles, uninterruptible power supplies, power grid, renewable energy, consumer electronics, and industry^11^,^12^,^13^. Small-scale supercapacitors can be integrated with microelectronic devices to work as stand-alone power sources or as efficient energy storage units complementing batteries and energy harvesters, leading to wider use of these devices in many industries^14^. To a great extent, the performance of supercapacitors depends on the electrode material and structure which has been generally categorized into two major classes^15^: (1) pseudo-capacitive materials such as metal hydroxides, transition metal oxides and conducting polymers; (2) composite materials in which charging-discharging follows an electric double-layer mechanism. Recently, composites materials possessing both of electric double-layer and pseudo-capacitive characteristics received extensive concern^16^. Carbon materials, such as activated carbon, carbon fiber, carbon cloth, carbon nanotube, graphene, etc. are applied to the electrodes for their significant electrical conductivity and electrochemical stability^17^,^18^,^19^. However, such carbon materials are of high cost or nanotoxicity, which limit their wide applications. Therefore, developing lower cost carbon materials with higher specific capacitance and promoting electrochemical properties is perceptibly attractive^20^.

One of such candidates to modify low grade carbon material into high performance electrode material for supercapacitors is adding metal oxide, especially transition metal oxides such as MnO_2_, NiO, Co_3_O_4_, V_2_O_5_, and CuO^21^,^22^,^23^. Among those metal oxides, NiO is considered to be one of the most attractive materials due to its good redox activity and high theoretical specific capacitance (2584 F g^−1^)^24^. For high-performance supercapacitors, a wide variety of hierarchical NiO micro/nanostructures, including nanosheets, nanotubes, hollow microspheres, flower-like microspheres have been investigated^24^,^25^,^26^,^27^. Although great progress has been made, it is still a great challenge to make a balance between excellent pseudo capacitive performance and simplicity of the synthetic pathway of NiO.

We hypothesized that the Ni-loaded biochar could be further reused as a potential material for generation of supercapacitors with both pseudo capacitive and electric double-layer properties. These may not only solve the Ni-loaded sorbent waste disposal but also expand a significant utilization of biochar as an effective energy storage device, both of which has great environmental significance. Therefore, the first part of this work was obtaining Ni-loaded biochar; the second part focused on preparation of supercapacitors by using the obtained Ni-loaded biochar, and the last part elucidated the underlying mechanisms for electrochemical performance of the biochar supercapcitor.

## Results and Discussion

### Characterization of biochar and Ni-loaded biochar

The contents of C in the original DM and SS biochars were 65.1% and 77.6%, respectively (Table 1), which was within the normal range of C for biochar^28^. After Ni sorption, C in the DM and SS biochars were reduced, especially in the biochars with microwave treatment, C was reduced to 55.3%. In correspondence, O increased from 8.10% and 16.6% to 35.2% and 39.3% for DM-Ni-MW and SS-Ni-MW, respectively. There was no detectable Ni in both original DM and SS biochars, however, after sorption both biochars contained 1.60% and 1.19% Ni, respectively (Table 1). This further demonstrated that biochar is an effective sorbent for sorption of Ni^5^. With the microwave treatment, Ni concentrations in the DM-Ni-MW and SS-Ni-MW biochar were elevated to as high as 3.85% and 3.26%, respectively (Table 1).

**Figure 1:**
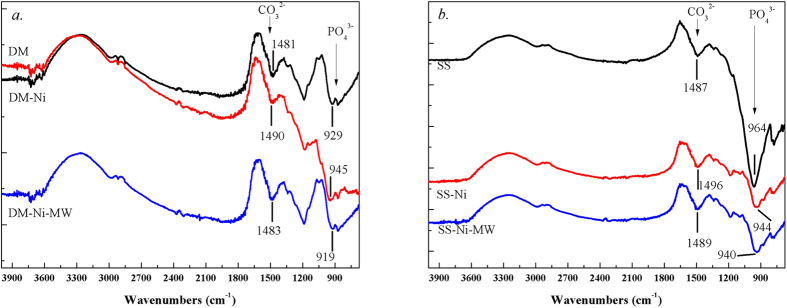
FTIR spectra of DM (a) and SS (b) biochar samples before and after Ni sorption.

The FTIR spectra showed that after Ni was sorbed, the peak of CO_3_^2−^ at 1480 cm^−1^ 29 in the DM and SS biochars shifted from 1481 cm^−1^ to 1490 cm^−1^, and from 1487 cm^−1^ to 1496 cm^−1^, respectively (Fig. 1). The results indicated that the sorption of Ni on biochar was probably through the combination of Ni^2+^ and CO_3_^2−^, possibly present as NiCO_3_. In addition, strong and wide peaks at 960–920 cm^−1^ assigned to PO_4_^3^ were observed in both biochars, and Ni-loaded induced the peak PO_4_^3−^ in DM and SS biochars shifted from 929 cm^−1^ to 945 cm^−1^ and from 964 cm^−1^ to 944 cm^−1^, respectively. The observation indicated a possible formation of Ni_3_(PO_4_)_2_ during the sorption of Ni on biochar. Many studies proved that sorption of heavy metals by biochar could be attributed to their precipitation with biochar-inherent anion ions such as CO_3_^2−^ and PO_4_^3−^ 30,31,32 Complexation of Ni with O-containing groups is also possible although not significant shift of the peaks of O-containing group, especially the peaks of 3000–3600 cm^−1^ associated with phenolic-OH, was observed before and after Ni sorption (Fig. 1).

The specific surface area of original DM and SS biochars were 37.4 m^2^ g^−1^ and 47.0 m^2^ g^−1^ respectively, which were within the general range of biochar as reported before^33^,^34^. Ni-loaded had little influence on the surface area. However, after microwave treatment, the specific surface areas of both Ni-loaded DM and SS biochars were reduced (Table 1). These may be caused by the newly generated fine particles which could block the pores, resulting in the decrease in surface area^35^,^36^. The consistent decrease in the micro-, meso-, and macro-pore volume of biochar further supported the possibility of pore blocking and correspondingly decrease of the surface area.

### Oxidation of biochars with microwave treatment

**Figure 2:**
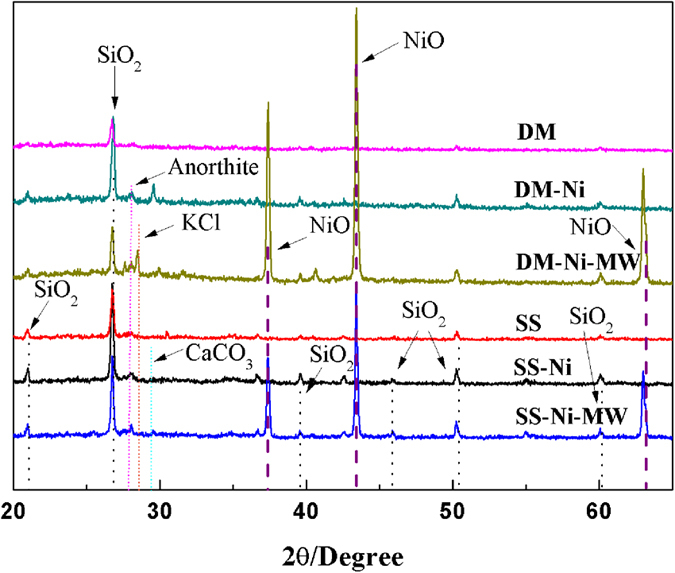
XRD patterns of DM and SS biochars, in comparison with the standard NiO.

**Figure 3:**
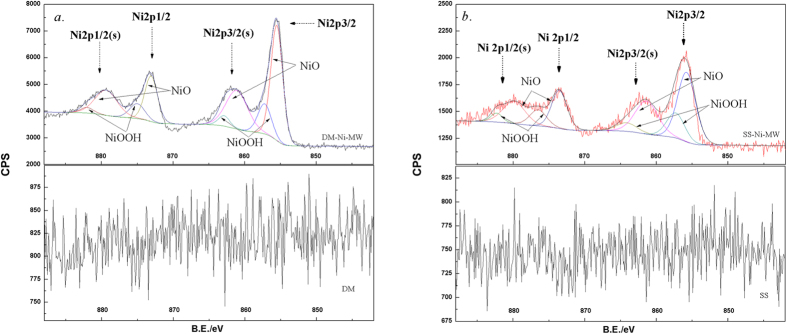
Ni binding energy of biochars ((**a**) DM, and (**b**) SS).

Microwave treatment could capture/transfer more O element on biochar samples, resulting in oxidation of the surface groups on samples^37^,^38^,^39^. Microwave irradiation activates substances by three mechanisms which are dipole polarization, ionic conduction, and the combination of two which was called interfacial polarization. It shortened reaction time by interacting with the reaction mixtures on a molecular level, leading to an accelerated rate of reaction and hence the oxidation of organic or inorganic groups^40^,^41^. By comparing Ni-loaded biochars before and after MW treatment, the peaks of PO_4_^3−^ remained less changed, mainly due to the higher stability of Ni_3_(PO_4_)_2_. However, the peaks of CO_3_^2−^ in both Ni-loaded biochars shifted back (from 1490 cm^−1^ to 1483 cm^−1^ for DM, and 1496 cm^−1^ to 1489 cm^−1^ for SS), indicating a possible transformation of NiCO_3_ during MW treatment. XRD analysis evidenced the presence of oxidized Ni particles, i.e., formation of NiO at the peaks of 2θ degree = 37°, 44.5°, and 66° 42 in both SS-Ni-MW and DM-Ni-MW biochars (Fig. 2). No peaks of NiCO_3_ or Ni_3_(PO_4_)_2_ were shown in both DM-Ni and SS-Ni samples, probably because of low concentration. The Ni concentrations in DM-Ni and SS-Ni were 1.60% and 1.19% (Table 1), below the detection limit of XRD. In addition to NiO, another Ni oxide, NiOOH was also formed which was confirmed by XPS analysis (Fig. 3). For both DM-Ni-MW and SS-Ni-MW biochars, the Ni2p1/2 and Ni2p3/2 photopeaks located at 873 and 856 eV with their satellites of 879 and 862 eV were assigned to Ni oxide-bearing compounds NiOOH and NiO, respectively^43^. The formation of NiO is important as it could improve specific capacitance^44^. Note that the noise signals in original DM and SS biochar are due to the presence of limited Ni.

Those results showed that the microwave treatment succeeded in the transformation of sorbed Ni on the biochar samples into Ni oxide particles. The transformation of Ni during the whole process from sorption to microwave treatment could be proposed as follows:

Sorption:


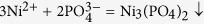



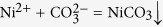



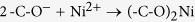



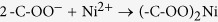


Microwave activation:


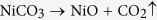



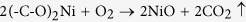



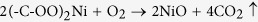



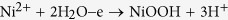


C and O peaks in both biochars were also analyzed, showing the existence of a variety of C groups (Figure S2 and Table S2): aromatic C at 284.71–284.75 eV, hydroxyl C at 285.56–286.16 eV, quinine C at 287.10–49 eV, and carboxyl/ester C at 288.03–289.07 eV. All chemical C bond shifted less after sorption and MW oxidation (Figure S2), which could be explained by the high stability of carbon framework. The O-containing groups are shown in Figure S2 and summarized in Table S2-quinine O at 531.11–531.64 eV, carboxyl/ester O at 532.00–533.54 eV, and anhydride O at 532.84–533.40^45^. Microwave oxidation induced all O-containing groups shift, especially carboxyl/ester O whose shift was much more obvious, it is probably due to break of (-C-OO)_2_Ni or (-C-O)_2_Ni. The increase in O-containing functional groups could contribute to the pseudo-capacitance^43^.

### Electrochemical characteristics of biochar supercapacitors

**Figure 4:**
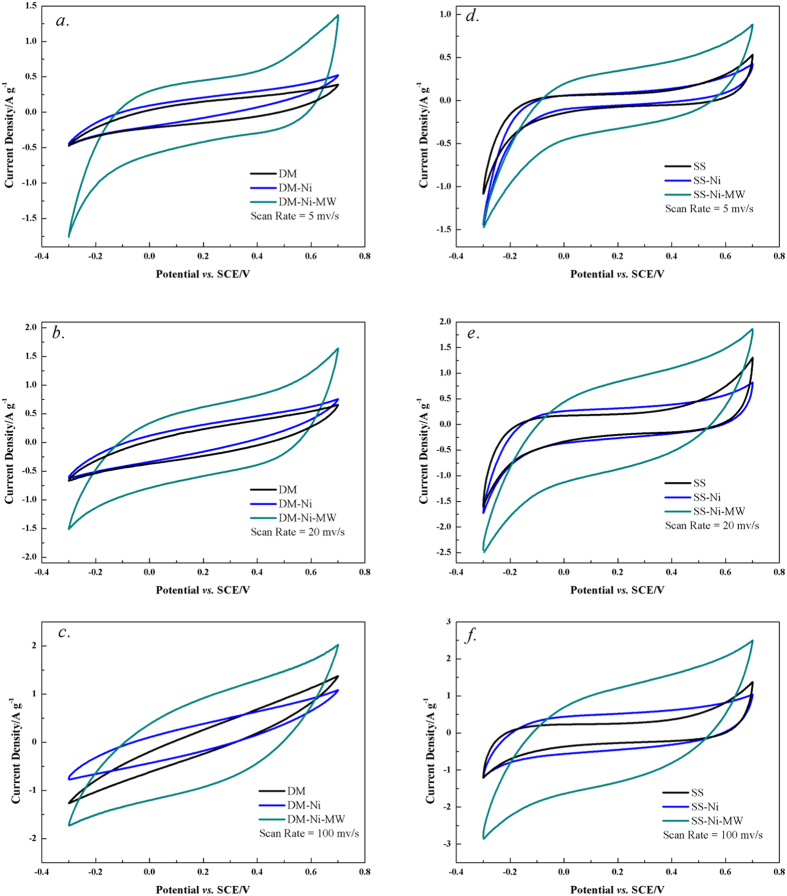
Cyclic voltammetry curves of all DM and SS biochar supercapacitors at different scan rates. (**a**) DM 5 mv/s, (**b**) DM 20 mv/s, (**c**) DM 100 mv/s, (**d**) SS 5 mv/s, (**e**) SS 20 mv/s, and (**f**) SS 100 mv/s.

The cyclic voltammetry curves for all biochar supercapacitors are shown in Fig. 4. Diagrams for all samples show typical electrochemical double layer (EDL) characteristics between −0.3 and +0.5 V versus Hg/HgO (saturated calomel electrode, SCE) in 0.5 M KOH with a pH value of 8.2 (0.43 V–1.23 V *vs* RHE). The O reduction wave between −0.1 and +0.5 V corresponded to the reduction of O-containing functional groups such as quinone and ketone as discussed before. The voltammograms obtained from the DM-Ni-MW and SS-Ni-MW biochars had a pseudo-rectangular-like shape which also increased with increasing san rate (Fig. 4). The pseudo-rectangular-like shape is close to the ideal EDL capacitive response^46^. This is attributed to the longer time available for electron to access the micropore of biochar due to the presence of NiO or NiOOH.

Microwave activated Ni-loaded biochar supercapacitor showed the highest specific capacitance, compared to the original biochar and Ni-loaded supercapacitors (Table 2). The capacitance of DM-Ni-MW and SS-Ni-MW were 123 F g^−1^ and 100 F g^−1^, increased by 2.14 and 2.01 times, respectively, compared to their original biochar supercapacitors (39.1 F g^−1^ and 33.2 F g^−1^, respectively). It was found that both double-layer capacitance and Faradic pseudo-capacitance existed in the metal oxide/carbon composites. The amount of electric charge stored in double-layer depends primarily on the electrode surface. Obviously, the surface area of supercapacitors was not responsible for the increase in the capacitance since the surface area decreased from the original biochar to microwave-treated Ni-loaded biochar (Table 1). The enhanced capacitance of activated Ni-loaded biochar supercapacitor was most likely attributed to the presence of NiO^24^,^27^. More similar work proved that metal oxides such as NiO and Co_2_O_3_ could enhance capacitance of supercapacitors^21^.

The Ni oxides particles in the electrochemistry system could undergo the following reaction to donate or accept electrons^44^:


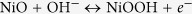


**Figure 5:**
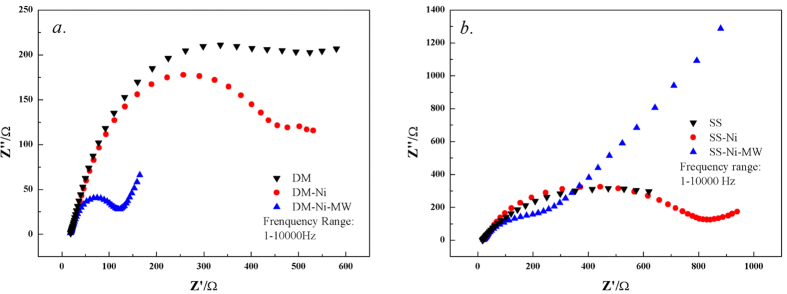
Electrochemical impedance spectroscopy of all DM (**a**) and SS (**b**) biochar supercapacitors. Scan voltage: 10 mv.

Figure 5 shows the electrochemical impedance spectroscopy (EIS) of all biochar supercapacitors. The calculated resistance values by fitting Re(Z) are listed in Table 2. All Re(Z) data showed that the Ni-loaded and MW treatment process led to the decrease of resistance. The Re(Z) in DM-Ni-MW and SS-Ni-MW were reduced by 3.35 and 2.49 times, compared to the original DM and SS biochar supercapacitors (Table 2). Resistance decrease was because of more conductor in the samples with the Ni-loaded. With the further MW treatment, the inner structure become more highly ordered and porous and NiO/NiOOH was formed, resulting in the easier conductor and correspondingly the decrease in the resistance. This result was similar to the other NiO/carbon based composite electrode in which formation of NiO has a significant promotion for electrochemical properties^43^.

After1000 charge-discharge cycles, the change in the specific capacitance was less, with only 3.5% and 4.5% lost for DM and SS biochar supercapacitors, respectively (Figure S5 and Table 2). It indicated that the high capacitance stability of biochar supercapacitors, especially for the activated Ni-loaded supercapacitors. The loss in the specific capacitance of DM-Ni-MW and SS-Ni-MW was only 1.6% and 1.8%, respectively (Table 2).

## Conclusions

This study showed that Ni-loaded dairy manure and sewage sludge biochars can be further reused for the supercapacitor production. Further treatment with microwave activation could increase the capacitance. The increase of capacitance in the Ni-loaded biochar supercapacitor following microwave treatment was mainly attributed to the conversion of Ni(II) into NiO and NiOOH. The biochar supercapacitors, especially microwave-treated Ni-loaded biochar supercapacitor exhibited the high stability of specific capacitance, with less than 2% loss after 1000 charge-discharge cycles. To improve the specific capacitance, future study will be conducted to test more strong activation method for example, chemical oxidation and plasma oxidation. Note that single heavy metal Ni was used as an example by converting the Ni-loaded biochar into supercapacitor in this paper. In fact, the practical wastewater may contain multiple heavy metals such as Cu, Zn, etc. Sorption of multiple heavy metals could affect the properties of the loaded biochar as supercapacitors. In the future, an attempt will be made on the reuse way of exhausted biochars after sorbing multi-metals in practical wastewater and determination of the effect of co-adsorbed metals on the properties of the biochar as supercapacitors.

This is the first study that reports reuse of transition metal-adsorbed-biochar. More importantly, the way created in this work may not only solve the heavy metal-laden sorbent waste disposal but also expand utilization of biochar as an effective energy storage device, both of which has a great significance of environmental sustainability.

## Materials and Methods

### Preparation of biochar and Ni-loaded biochar

Two waste biomasses, sewage sludge and dairy manure were collected from a wastewater treatment plant and a livestock farm in Shanghai, respectively. The waste biomasses were then air-dried to constant weight. The details for biochar production were described in a previous work^49^. Specifically, the dried manure and sewage sludge was put in a stainless steel reactor and heated in a Muffle Furnace under <0.5% O_2_ condition at 600 °C for 4 h. The solid residue left in the reactor after heating was C-rich material and called as biochar. The resulted biochar was ground in a ball-milling machine and passed through a 0.5-mm sieve for later experiment. Biochars derived from dairy manure and sewage sludge were referred to as DM and SS, respectively.

To obtain Ni-loaded biochar, the batch sorption experiment was performed. Specifically, 0.20 g biochar was added into 40 mL of 0–200 mg L^−1^ Ni(NO_3_)_2_ solution in a series of 50-mL polypropylene tubes. The mixture of biochar and Ni(NO_3_)_2_ solution was shaken at 240 rpm for 24 h under room temperature (20 ± 2 °C). After equilibrium, solution and solid were separated by centrifugation using 0.45-μm water-filtration-membrane. The filtrate was acidified to pH < 2.5 with concentrated HNO_3_ for Ni analysis by using flame atomic absorption spectrometry (FAAS, Zeeman Z 2000, Hitachi, Japan). The residue retained on the filter was Ni-loaded biochar and collected for further activation and fabrication of supercapacitors. For simplicity, Ni-loaded DM and SS biochars were referred to as DM-Ni and SS-Ni, respectively.

### Activation of Ni-loaded biochar

In order to promote electrochemical property of biochar, microwave (MW) oxidation were employed to activate Ni-loaded biochar. It has been reported that MW oxidation is an effective method for activation of carbon materials, and the details for activation was described previously^37^,^38^,^39^,^40^. Specifically, about 3.0 g Ni-loaded biochar (DM-Ni and SS-Ni) was put in a PTFE cube with 5.0 ml deionized water, and heated in microwave oven under 1000 W power for 30 min. The DM-Ni and SS-Ni biochars activated by microwave oxidation were referred to as DM-Ni-MW and SS-Ni-MW, respectively.

### Characterization of biochar

The composition, structure, and porosity of all biochar samples were characterized by using a variety of analytical methods. Elemental composition of C, H, and N in all biochars was analyzed by using an element analyzer (Vario EL III, Elemental, Germany). The Brunauere-Emmete-Teller (BET) method was used to measure the specific surface area, micropore volume, and mesopore volume of all biochars using BET-N_2_ analyzer (JW-BK222, JWGB, China) (Figure S4). To explore the mechanism of Ni sorption, Ni transformation, and interaction between Ni and biochar, X-ray photoelectron spectroscopy (XPS, AXIS Ultra DLD, Shimadzu Kratos, Japan), X-ray diffraction (XRD, D/max-2200/PC, Rigaku, Japan) and Fourier transform infrared spectrometry (FTIR, IR Prestige 21 FTIR, Shimadzu, Japan) were employed. For the sample preparation, all biochars were sieved through 170-mesh to remove most of bigger-size char, which was attempted to concentrate Ni for detection.

### Fabrication of biochar supercapacitors

The supercapacitors were prepared from original, Ni-loaded, and activated Ni-loaded biochars by employing a dry-mixture method^50^: Painting biochar slurry onto stainless steel wire, and then cut it into rectangle shape with a size of 10 mm × 5 mm × 0.3 mm. The biochar slurry was prepared by mixing with Polytetrafluoroethylene (PTFE) at a 1.5:8.5 mass ratio of PTFE to biochar. To form the electrode, the biochar slurry was pressed onto the stainless steel wire for 10 min under 6 × 10^5^ pa force. The active electrode mass loaded on each stainless steel was about 0.5 mg for DM and 0.1 mg for SS, and the resulted thickness of an electrode was 700 μm, which contains a layer of biochar (300 μm) and the other layer of stainless steel (400 μm).

### Electrochemical characterization of supercapacitors

The inorganic electrolyte used was 0.5 M KOH. The details for electrode preparation are described elsewhere^47^. The electrical conductivity of biochar supercapacitors was measured by using a three-probe method. Cyclic voltammetry measurement was performed by using a potentiostat^48^. The impedance of the fabricated supercapacitors was measured by using an impedance spectroscopy, with a frequency range of 1 Hz –10 KHz and potential amplitude of 10 mV. The specific capacitance was calculated from cyclic voltammetry characteristics and expressed in Farads per gram of biochar deposited on the stainless steel. The calculation equation is given below:


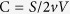


*S* is the integrated area of cyclic voltammetry, *v* is the scanning rate, and *V* is the scanning potential range.

## Additional Information

**How to cite this article:** Wang, Y. *et al*. Converting Ni-loaded biochars into supercapacitors: Implication on the reuse of exhausted carbonaceous sorbents. *Sci. Rep.* **7**, 41523; doi: 10.1038/srep41523 (2017).

**Publisher's note:** Springer Nature remains neutral with regard to jurisdictional claims in published maps and institutional affiliations.

## Supplementary Material

Supporting Information

## Figures and Tables

**Table 1:** Main properties of original biochar and Ni-loaded biochar.

Biochar	Treatment	C%	H%	O%	Ni mg kg^−1^	SA m^2^ g^−1^	Micropore volume cm^3^ g^−1^	Mesopore volume cm^3^ g^−1^	Macropore volume cm^3^ g^−1^
DM	Original	77.6	2.30	8.10	BDL	37.4	0.017	0.018	0.024
DM-Ni	Ni-loaded	75.2	2.17	8.05	16,000	36.1	0.012	0.017	0.021
DM-Ni-MW	Microwave	55.3	1.10	35.2	55,700	29.8	0.013	0.015	0.018
SS	Original	65.1	4.42	16.6	BDL	47.0	0.012	0.014	0.008
SS-Ni	Ni-loaded	63.1	4.51	16.5	11,900	45.2	0.011	0.013	0.007
SS-Ni-MW	Microwave	43.1	3.31	39.3	41,300	31.5	0.009	0.012	0.009

**Table 2:** Specific capacitance, resistance values, and specific capacitance loss (%) of DM and SS supercapacitors.

	Dairy Manure Biochar			Sewage Sludge Biochar		
	DM	DM-Ni	DM-Ni-MW	SS	SS-Ni	SS-Ni-MW
Capacitance/F g^−1^ (5 mv/s)	39.1	42.7	123	33.2	36.9	100
Re(Z′)/Ω	610	562	140	947	902	271
Specific capacitance loss %	3.1	2.2	1.6	4.5	3.7	1.8
