# Supplementary material for: Converting Ni-loaded biochars into supercapacitors: Implication on the reuse of exhausted carbonaceous sorbents
Source: Sci Rep. 2017 Jan 27;7:41523. doi: 10.1038/srep41523 (PMC5269738; doi:10.1038/srep41523)
Supplement: Supporting Information [file srep41523-s1.doc]

**Supporting Information**

**Converting Ni-loaded biochars into supercapacitors: Implication on the reuse of exhausted carbonaceous sorbents**

Yifan Wang, Yue Zhang, Lei Pei, Diwen Ying, Xiaoyun Xu, Ling Zhao, Jinping Jia, Xinde Cao*

School of Environmental Science and Engineering, Shanghai Jiao Tong University

Shanghai 200240, China

* Corresponding author. Tel: +86-21-54743926, e-mail: xdcao@sjtu.edu.cn

8 pages include 5 figures and 2 tables


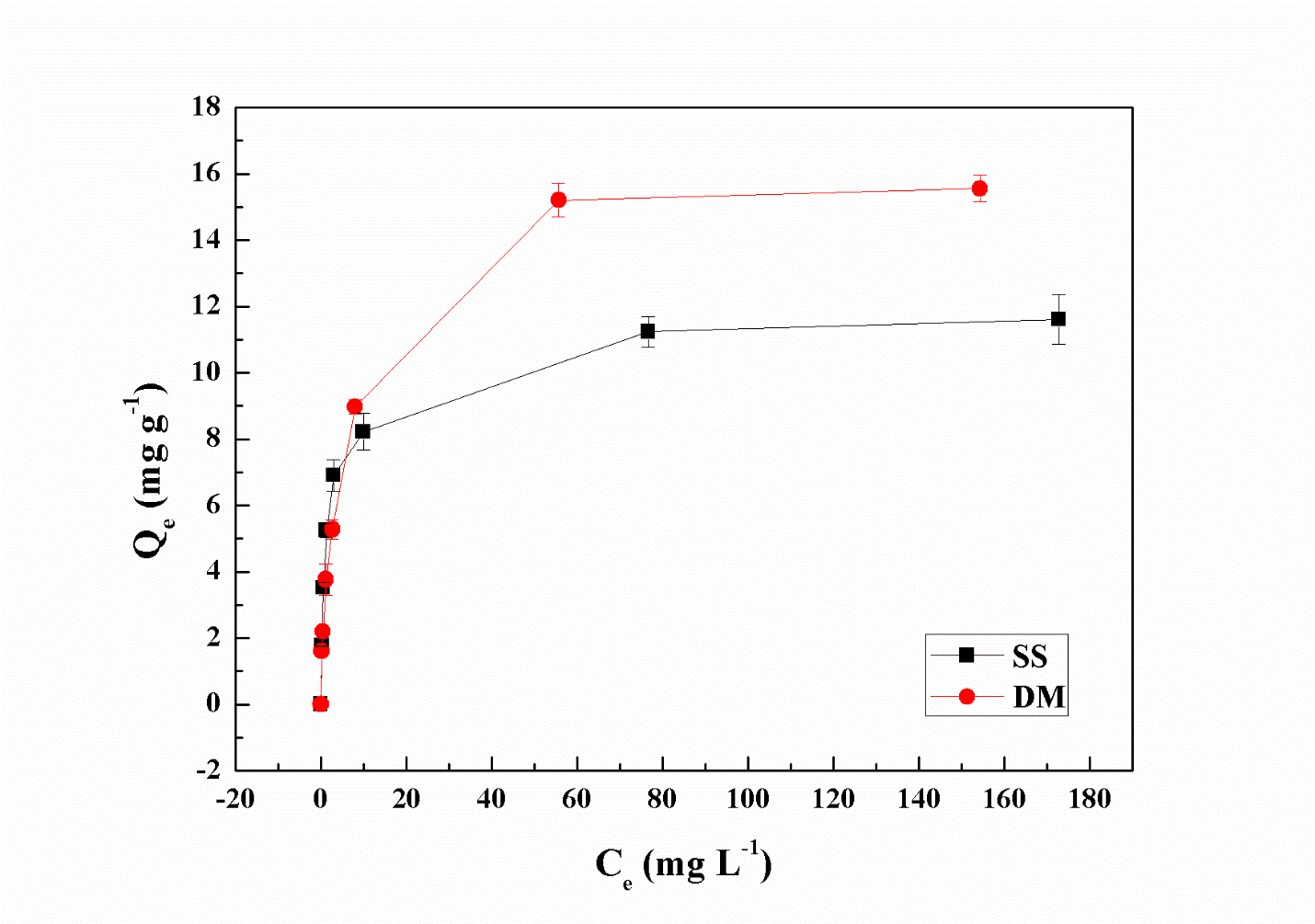


**Figure S1.** Sorption isotherm of Ni by original DM and SS biochars


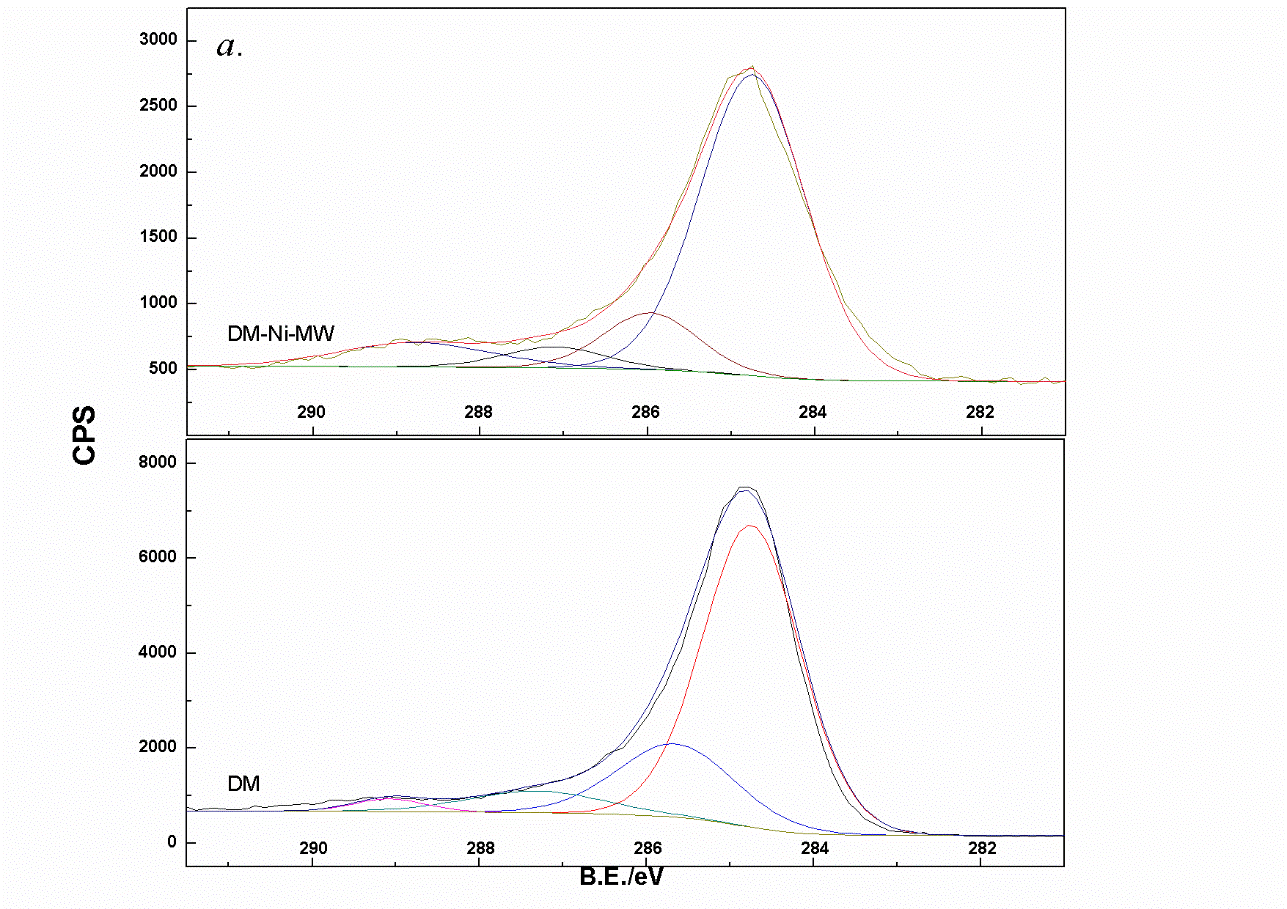


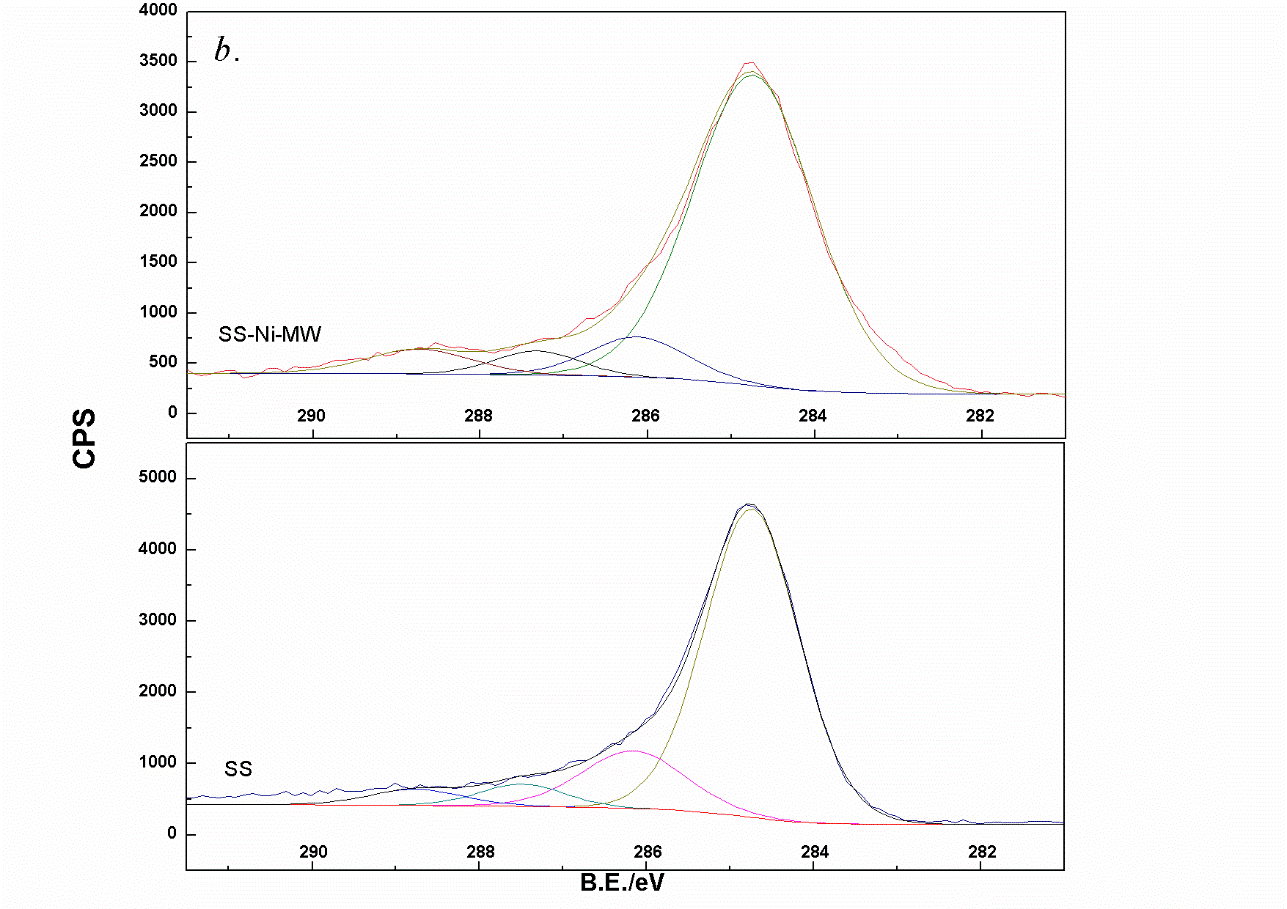


**Figure S2.** C binding energy of biochars (a. DM, and b. SS)


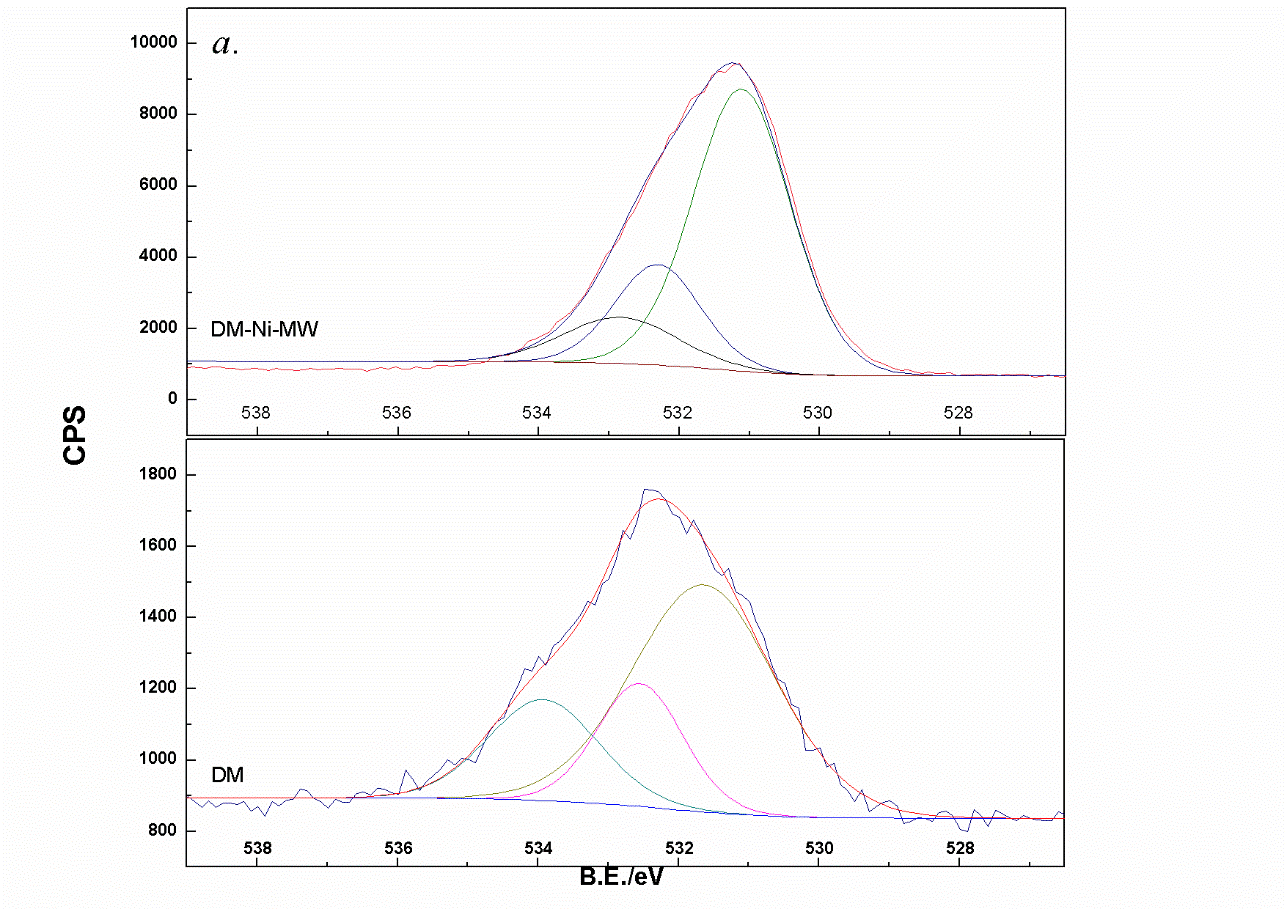

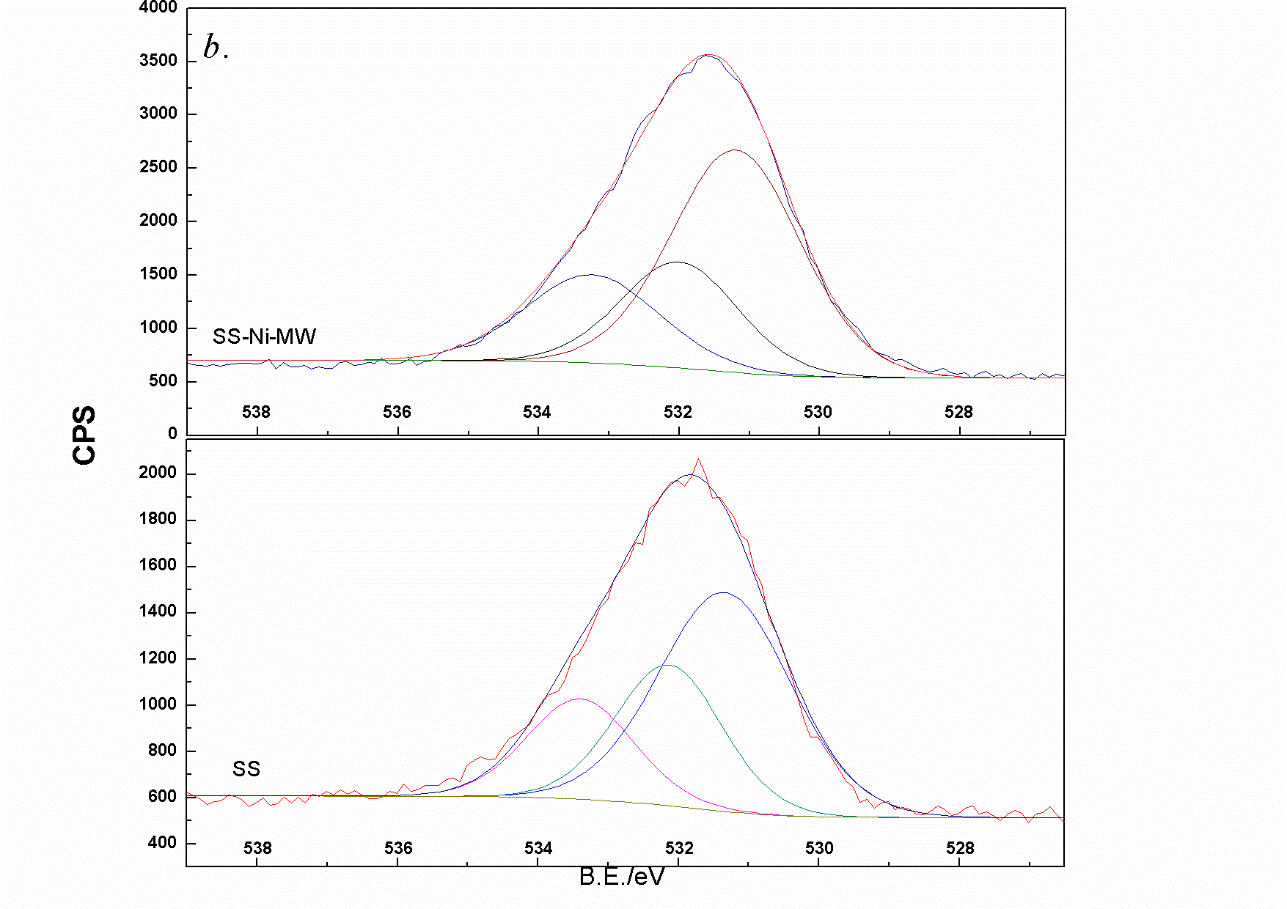


**Figure S3.** O binding energy of biochars (a. DM, and b. SS)

**
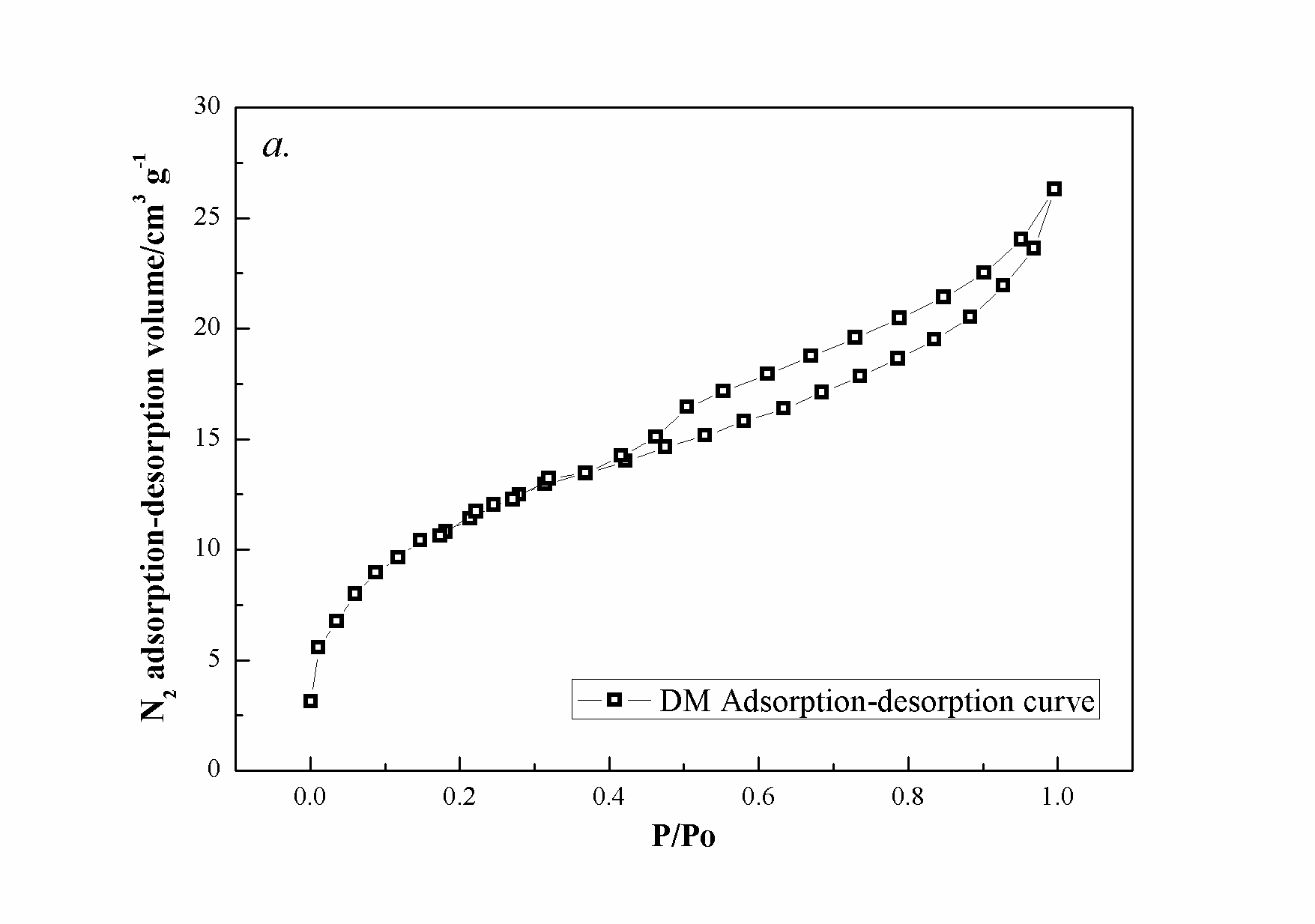
** **
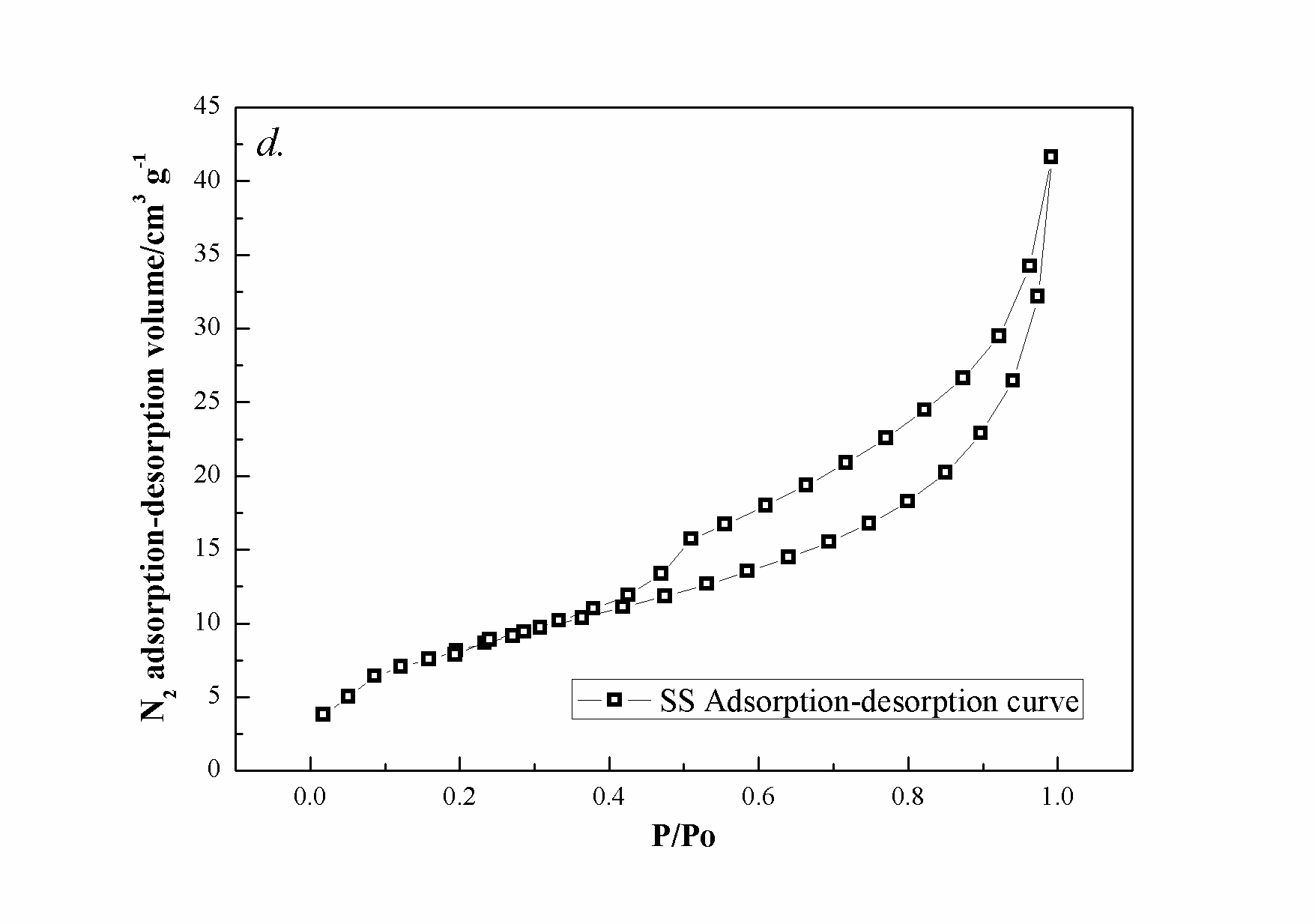
**

**
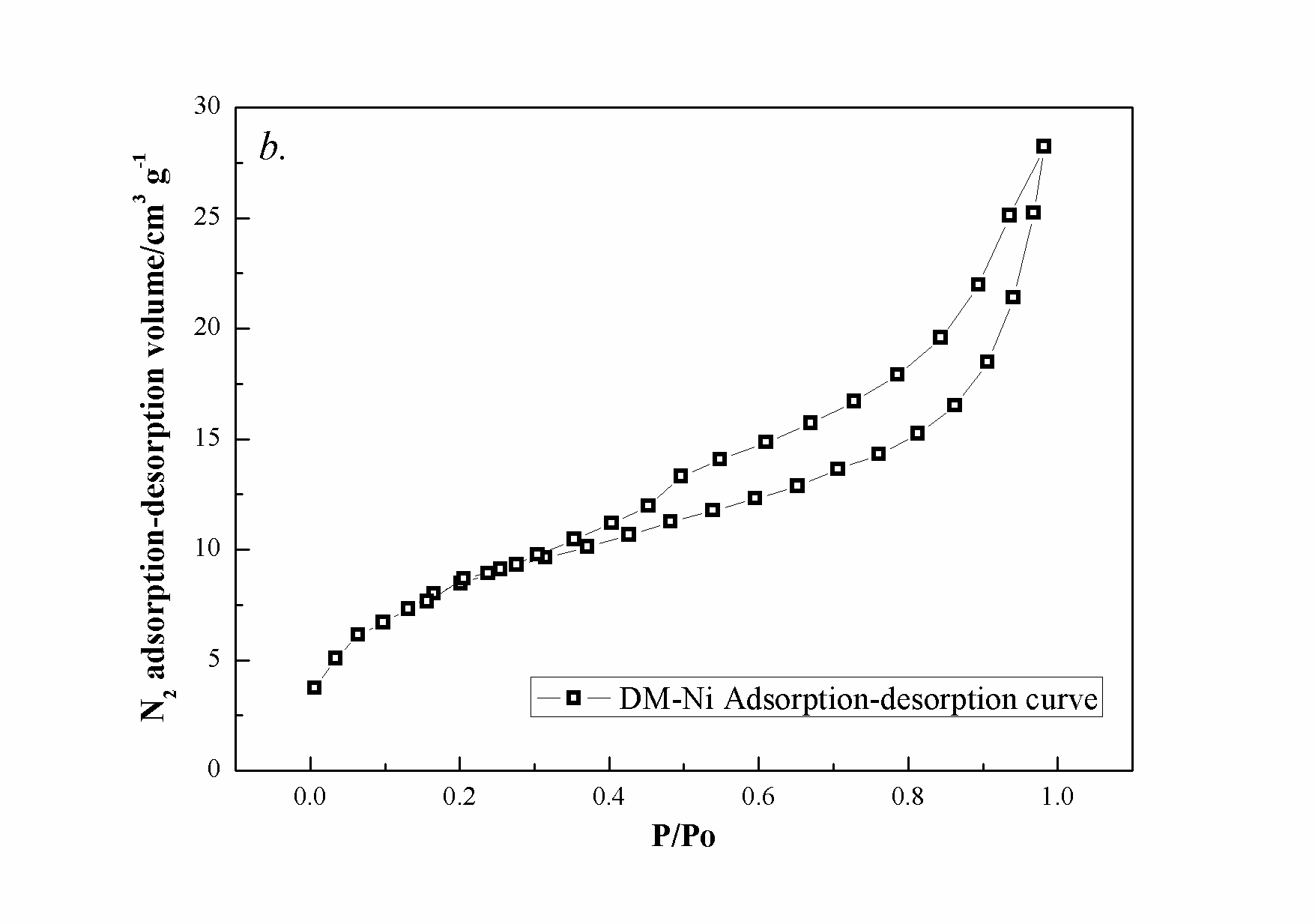
** **
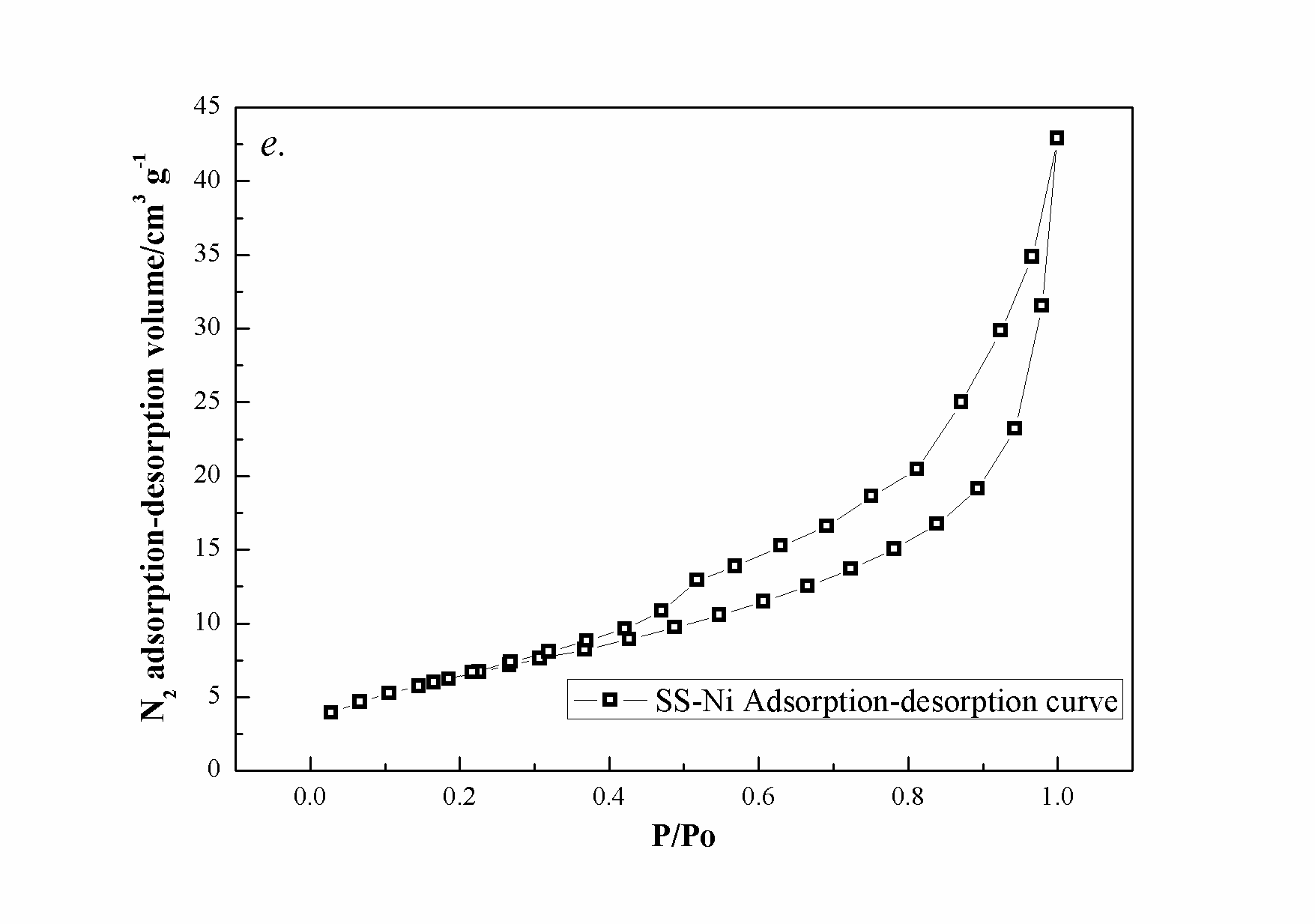
**

**
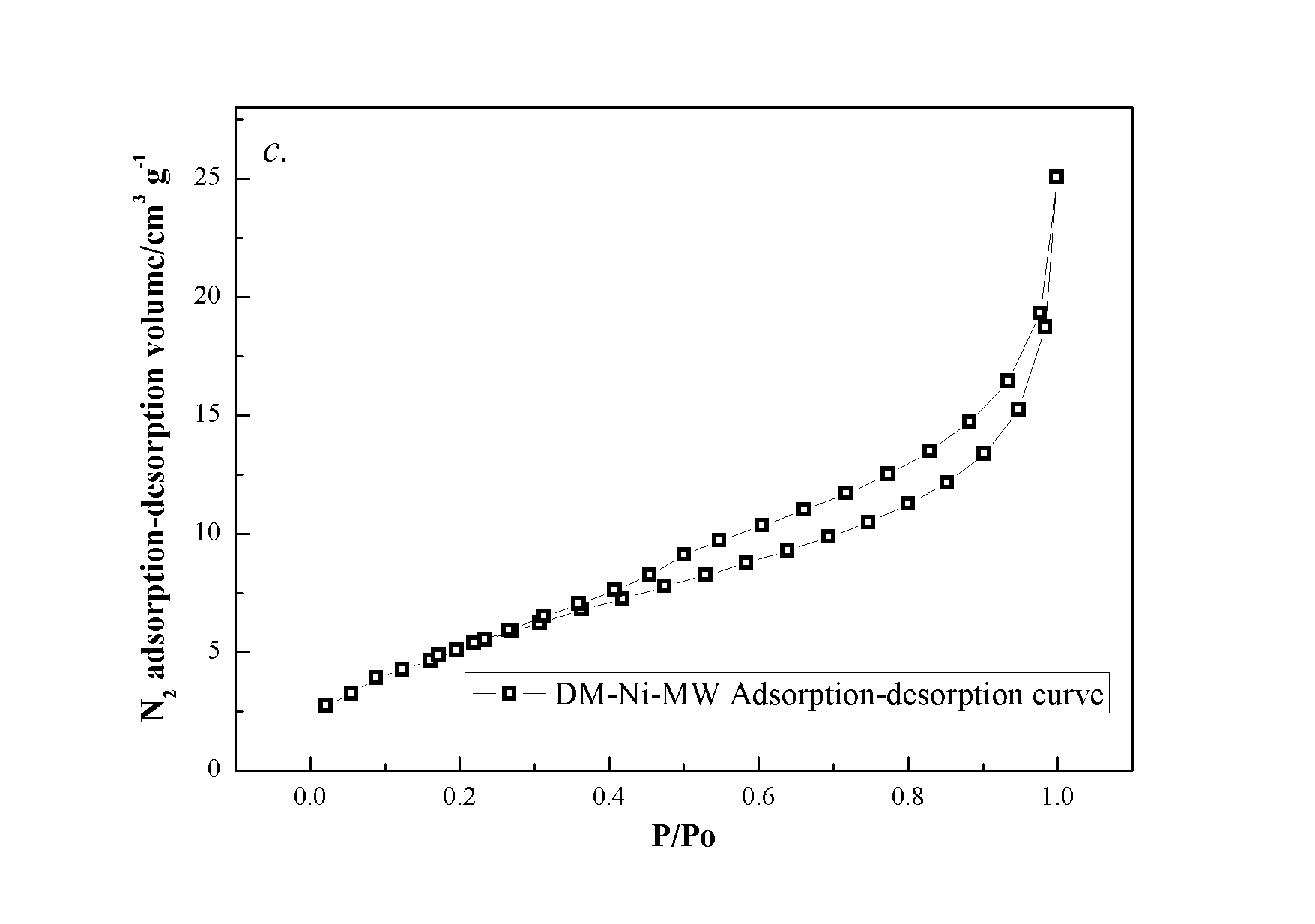
** **
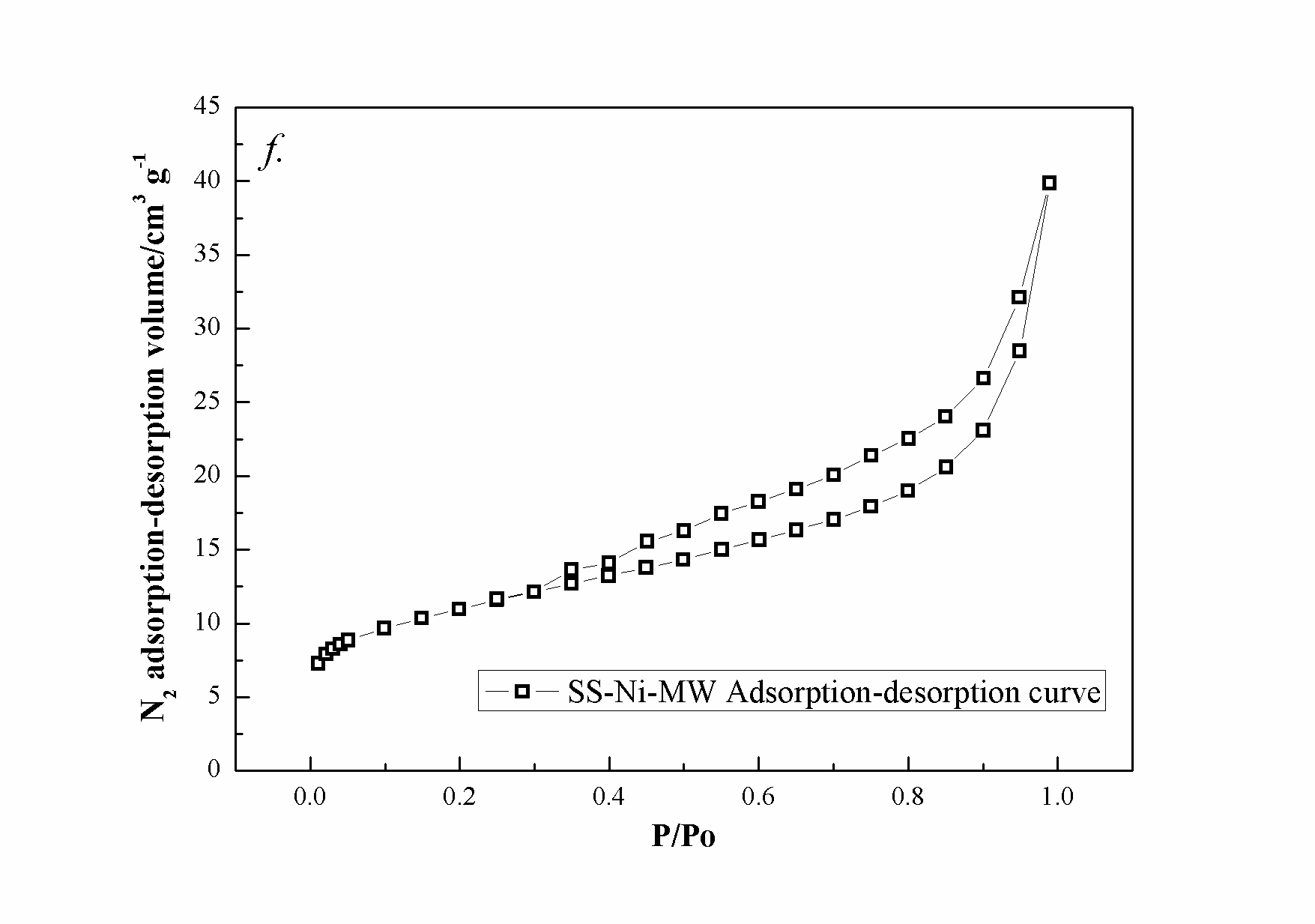
**

**Figure S4.** N_2_ adsorption-desorption curve of biochar samples (*a*. DM, *b*. DM-Ni, *c*. DM-Ni-MW, *d*. SS, *e*. SS-Ni, *f*. SS-Ni-MW)


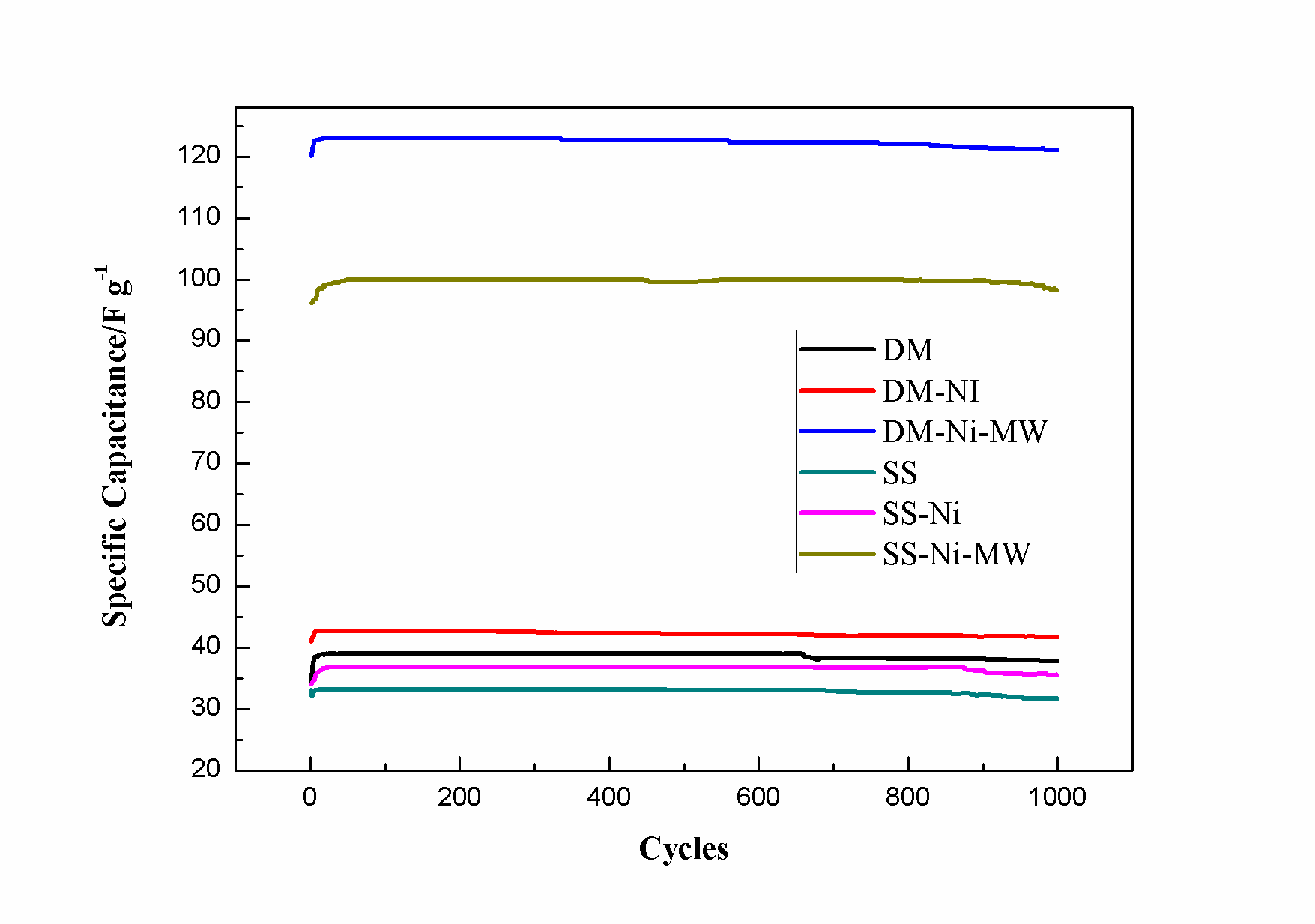


**Figure S5.** Specific capacitance *vs*. number of charge-discharge cycles for DM & SS

**Table S1.** Isotherm parameters of Ni sorption by DM and SS biochars

| Adsorbent | Langmuir | | | Freundlich | | |
| --- | --- | --- | --- | --- | --- | --- |
|  | *Q_m_*(mg g^-1^) | *K _L_*(L mg^-1^) | *R^2^* | *K_f_*(mg g^-1^) | 1/*n* | *R^2^* |
| DM | 16.0± 0.39 | 2.13 ± 0.09 | 0.884 | 3.67 ± 0.27 | 0.16 ± 0.05 | 0.967 |
| SS | 11.9 ± 0.21 | 5.76 ± 0.28 | 0.994 | 3.55 ± 0.23 | 0.12 ± 0.05 | 0.843 |

**Table S2.** XPS peak positions (eV) of C, O, and Ni in the original biochar and MW-treated Ni-loaded biochar

|  |  | | | |
| --- | --- | --- | --- | --- |
|  | DM | DM-Ni-MW | SS | SS-Ni-MW |
| C | 284.75 | 284.74 | 284.71 | 284.74 |
|  | 285.56 | 285.97 | 286.16 | 286.12 |
|  | 287.10 | 287.12 | 287.49 | 287.32 |
|  | 289.07 | 288.78 | 288.75 | 288.03 |
|  |  |  |  |  |
| O | 531.64 | 531.11 | 531.34 | 531.19 |
|  | 532.54 | 532.30 | 532.14 | 532.00 |
|  | 533.93 | 532.84 | 533.40 | 533.25 |
|  |  |  |  |  |
| Ni | N. A. | 855.52 | N. A. | 855.78 |
|  |  | 857.22 |  | 857.32 |
|  |  | 862.89 |  | 861.39 |
|  |  | 861.11 |  | 863.15 |
|  |  | 873.07 |  | 873.57 |
|  |  | 875.01 |  | 876.52 |
|  |  | 882.00 |  | 879.89 |
|  |  | 879.36 |  | 882.33 |
